# Treatment burden and health-related quality of life of patients with multimorbidity: a cross-sectional study

**DOI:** 10.1007/s11136-023-03473-3

**Published:** 2023-07-05

**Authors:** Eyob Alemayehu Gebreyohannes, Begashaw Melaku Gebresillassie, Frehiwot Mulugeta, Etsegenet Dessu, Tamrat Befekadu Abebe

**Affiliations:** 1 Department of Clinical Pharmacy, School of Pharmacy, University of Gondar, Gondar, Ethiopia; 2 Division of Pharmacy, School of Allied Health, University of Western Australia, Perth, WA Australia; 3 Geospatial Health and Development, Telethon Kids Institute, Perth, WA Australia; 4 Center for Medicine Use and Safety, Faculty of Pharmacy and Pharmaceutical Sciences, Monash University, Clayton, VIC Australia

**Keywords:** Cross-sectional studies, Multimorbidity, Treatment burden, Patient-reported outcome measures, Quality of life, Polypharmacy

## Abstract

**Purpose:**

The aim of this study was to investigate treatment burden and its relationship with health-related quality of life (HRQoL) among patients with multimorbidity (two or more chronic diseases) who were taking prescription medications and attending the outpatient department of the University of Gondar Comprehensive Specialized Teaching Hospital.

**Methods:**

A cross-sectional study was conducted between March 2019 and July 2019. Treatment burden was measured using the Multimorbidity Treatment Burden Questionnaire (MTBQ), while HRQoL was captured using the Euroqol-5-dimensions-5-Levels (EQ-5D-5L).

**Results:**

A total of 423 patients participated in the study. The mean global MTBQ, EQ-5D index, and EQ-VAS scores were 39.35 (± 22.16), 0.83 (± 0.20), and 67.32 (± 18.51), respectively. Significant differences were observed in the mean EQ-5D-Index (F [2, 81.88] 33.1) and EQ-VAS (visual analogue scale) scores (F [2, 75.48] = 72.87) among the treatment burden groups. Follow up post-hoc analyses demonstrated significant mean differences in EQ-VAS scores across the treatment burden groups and in EQ-5D index between the no/low treatment burden and high treatment burden, as well as between the medium treatment burden and high treatment burden. In the multivariate linear regression model, every one SD increase in the global MTBQ score (i.e., 22.16) was associated with a decline of 0.08 in the EQ-5D index (β − 0.38, 95%CI − 0.48,  − 0.28), as well as a reduction of 9.4 in the EQ-VAS score (β − 0.51, 95%CI -0.60, − 0.42).

**Conclusion:**

Treatment burden was inversely associated with HRQoL. Health care providers should be conscious in balancing treatment exposure with patients’ HRQoL.

**Supplementary Information:**

The online version contains supplementary material available at 10.1007/s11136-023-03473-3.

## Plain Language Summary

This study investigated how common treatment burden is and its impact on the quality of life of patients with multiple chronic diseases who take prescription medications and receive outpatient care at the University of Gondar Comprehensive Specialized Teaching Hospital. In this study, we surveyed 423 patients, reviewed their medical records, and asked them about the difficulties they faced in managing their treatments and their overall quality of life. The results showed that patients who experienced a higher treatment burden had lower quality of life compared to those with a lower treatment burden. Specifically, the patients who reported more challenges in managing their treatments had lower scores on measures of their overall well-being and quality of life. It is important to find a balance between providing necessary treatments and ensuring that patients can still enjoy a good quality of life.

## Introduction

Multimorbidity refers to the co-occurrence of two or more chronic diseases in an individual. [1] This condition is becoming more prevalent due to the increasing elderly population, which is a result of people living longer. [2] Low- and middle-income countries (LMICs) are disproportionately affected by the burden of chronic diseases and contribute to more than three-quarters of deaths from chronic diseases. [3] Multimorbidity is of particular concern in LMICs due to limited access to healthcare, low health literacy, and socioeconomic disparities. [4, 5] In addition to the burden associated with the symptoms of the diseases, the rise in the prevalence of multimorbidity has brought the additional burden of treatment to patients [6, 7]. Multimorbidity predisposes patients to complex medication regimens, polypharmacy, poor adherence, and medication-related adverse events. [1] As a result, patients with multimorbidity face a higher treatment burden than those with only a single disease. [8]

Treatment burden can be defined as “the workload of health care and its impact on patient functioning and well-being”. [9] It encompasses a number of aspects of patient care such as literacy and understanding medical information, taking multiple and complex medication regimens, monitoring, interacting with individuals including healthcare professionals, exercise and dietary changes, self-monitoring, numeracy, and others [6, 9–12]. Many patients living with chronic diseases report that sustaining the same level of commitment to managing their diseases for a long period of time is very difficult. [7] As such, patients experiencing treatment burden will have significantly diminished health-related quality of life (HRQoL). [6, 11] This comes as no surprise since patients experiencing treatment burden often feel overwhelmed with the amount of effort they have to put in managing their diseases. [9]

Not all patients perceive treatment burden the same way. Patients with multimorbidity are at a higher risk of treatment burden [10]. In addition, patients’ skills, cognitive and physical capabilities, the amount of social support they receive, their workload and ability to manage their workload, and financial status can influence how patients experience treatment burden. [10, 11] Further, younger age, low health literacy, and little overlap in managing multiple diseases, contribute to risk of a higher treatment burden in patients with multimorbidity. [8, 10]

According to a recent meta-analysis by Chowdhury et al., it was found that the pooled prevalence of multimorbidity worldwide stands at 37.2%. The analysis further indicated significant variations across income groups, with high-income countries having a prevalence of 38.6%, upper-middle income countries with 38.7%, and low-income countries with 32.1%. [13] Furthermore, there is a wide variation in the magnitude of multimorbidity within LMICs. A scoping review found that the prevalence of multimorbidity in adults aged 18 years and above ranged from 3.2% to 67.8% [14]. It also reported that the prevalence increases with advancing age. In Ethiopia, multimorbidity has become very prevalent. A study conducted in the current study setting reported that the prevalence of multimorbidity among patients with cardiovascular diseases was 44.6% [15]. With such a high prevalence of multimorbidity, it is expected that the treatment burden and HRQoL of patients can be significantly affected. This is especially very important in a country with poor health literacy [16] as studies show that individuals that have difficulties understanding health information are more likely to experience treatment burden. [8]

Although the connection between multimorbidity and treatment burden has been reported elsewhere [8], to the best of the authors’ knowledge and a literature search, the available literature on treatment burden and HRQoL in patients with multimorbidity in LMICs, especially in Ethiopia, is limited. Therefore, this study aims to fill the research gap in the existing literature on treatment burden and HRQoL in patients with multimorbidity in LMICs, especially focusing on Ethiopia, where a high burden of multimorbidity has been documented. [15, 17] Thereof, the objective of this study was to investigate treatment burden and its relationship with HRQoL among patients with multimorbidity.

## Methods

### Study design

The authors followed the STROBE cross-sectional reporting checklist to conduct an institution-based cross-sectional study at the Outpatient Department of the University of Gondar Comprehensive Specialized Teaching Hospital (UoGCSTH). [18] The healthcare system in Ethiopia is divided into three tiers: primary (consisting of primary hospitals, health centers, and health posts), secondary (includes general hospitals), and tertiary (includes specialized hospitals) levels of care. [19]

### Study population and setting

Adult patients (18 years and older) with multimorbidity were the primary interest of the current survey. Data were collected when patients came for routine check-ups or medication refills at the Outpatient Department in the UoGCSTH between March 2019 and July 2019. The UoGCSTH is a public hospital situated in the town of Gondar, which is located in the north-western part of Ethiopia, approximately 727 km away from the capital city of Addis Ababa. The hospital serves an estimated 13 million people in the surrounding catchment area. [20]

Potential participants were approached for participation after consecutively reviewing their medical records. To participate in the study, patients needed to be age ≥ 18 years old, diagnosed with at least two chronic diseases, and already on medical treatment for the relevant disorder. The World Health Organization defines chronic diseases, also known as non-communicable diseases, as diseases that tend to be of longer duration. [21] In this study, we defined chronic diseases as non-communicable diseases that lasted at least three months. Patients in emergency state conditions and those who had diseases that might preclude the administration of the instruments such as dementia or Alzheimer’s disease were excluded from the study.

### Instruments

Data on patients’ diseases and treatments were extracted from their medical records. To capture the objectives of our study, two instruments were selected to address the treatment burden and HRQoL. All data collection was conducted by two of the authors (FM and ED). While the two instruments used in the study were self-administered, for individuals who were unable to read or write, the same authors conducted face-to-face interviews. Throughout the study, the two data collectors checked the completeness of the data collection instruments after each questionnaire was completed to minimize the possibility of missing data. Both instruments were administered in the local language, Amharic (Appendix 1). The instrument for assessing HRQoL was available in Amharic. However, the instrument used to measure treatment burden was initially in English (Appendix 2) and was translated to Amharic by one of the authors (EAG). Another author (BMG) performed a back-translation to English to ensure accuracy and consistency. Any discrepancies were addressed through discussions among the research team.

### Treatment burden

The Multimorbidity Treatment Burden Questionnaire (MTBQ) is a 10-item instrument designed to capture the treatment burden in patients with multimorbidity. [22] MTBQ is validated to assess the effects of treatments that might influence the level of treatment burden in patients with multimorbidity. Each item provides Likert-like options: “extremely difficult”, “very difficult”, “quite difficult”, “a little difficult”, “not difficult” and “does not apply”, to grasp the level of difficulty patients experience in day-to-day activity while exposed to treatments. According to the scoring guideline for MTBQ [23], items with “does not apply” and “not difficult” score were recoded as 0; “a little difficult” score as 1; “quite difficult” score as 2; “very difficult” scored as 3; and “extremely difficult” scored as 4. After scoring each item, the mean was calculated and multiplied by 25 to give the global MTBQ score from 0 to 100 with the higher the score, the more the burden of treatment. The global MTBQ is interpreted as no burden (0), low burden (< 10), medium burden (10–< 22), and high burden (≥ 22). In the present study, MTBQ showed good reliability in terms of internal consistency (Cronbach’s alpha = 0.91).

### Health-related quality of life

A generic instrument, the Euroqol-5 dimensions-5-Levels (EQ-5D-5L), was employed to capture patients’ HRQoL. The instrument taps multiple aspects of patients’ HRQoL through items: mobility, self-care, usual activities, pain and discomfort, and anxiety and depression. Each dimension has five levels (1–5) ranging “no problems”, “slight problems”, “moderate problems”, “severe problems”, and “unable”. The last two levels of severities are rephrased as “severe pain” and “extreme pain” for the pain and discomfort dimension, and as “severely anxious” and “extremely anxious” for the anxiety and depression dimension. The combination of the dimensions will give 3125 (= 5^5^) possible value sets from 11,111 (full health) to 55,555 (extreme problems in all dimensions). The EQ-5D-index was calculated based on the EQ-5D-5L value set for the Ethiopian population [24]. The EQ-5D-index ranges from − 0.145 (worse than death, 55,555) to 0.9 (full health, 11,111). In addition, the EQ-5D-5L has an additional question called EQ-VAS (EQ-visual analogue scale), a vertically lined visual analogue scale ranging from 0 (worst health can be imagined) to 100 (best health can be imagined). Similar to MTBQ, the EQ-5D tool in the present study showed good reliability (Cronbach’s alpha = 0.87).

### Outcomes

The EQ-5D-Index and the EQ-VAS score were outcomes explored in the study. Outcomes from the EQ-5D-5L questionnaire were stratified by the level of treatment burden before any analysis was executed.

### Sample size

The G*Power software (version 3.1.9.4) was used to calculate the minimum sample size. Based on the sample size calculation for a one-way ANOVA, assuming a medium effect size (Cohen’s *f* = 0.25) [25], with 95% power, a significance level of 5%, and four independent groups representing different levels of treatment burden, a minimum sample size of 280 participants was required for this study.

### Statistical analysis

All statistical analyses were performed using R version 3.6.3. Continuous and categorical variables were presented as mean with standard deviation (SD) and number with proportion, respectively. Correlation of the global MTBQ score with EQ-5D index and EQ-VAS score was assessed by Spearman correlation. Differences in the EQ-5D outcomes (EQ-5D index and EQ-VAS score) across the treatment burden spectrum were analyzed by applying the Welch analysis of variance (Welch ANOVA). For the purpose of this analysis, treatment burden levels were reclassified as no or low burden, medium burden, and high burden. Whenever a significant difference was observed, the corresponding Games Howell post-hoc analysis was performed. Further association between the global MTBQ score and patients characteristics was examined by employing multivariate linear regression model adjusting for age, sex, number of comorbidities, and number of medications. Polypharmacy, the prescription of ≥ 5 medications per patient, was used as a criterion to categorize the number of medications. The EQ-5D outcomes in the current study exhibited a non-normal distribution. However, this characteristic was primarily attributed to the classification system’s design for setting severity levels and the corresponding weights used to calculate the index, rather than representing the true distribution of ill health. [26] Standardization of the continuous variables (age, EQ-5D index, EQ-VAS score, and global MTBQ score) was applied before the multivariate regression analysis by subtracting the mean value of the specific variable from the individual values then dividing it by the SD of the variable. Standardization of variables makes it easier to interpret the coefficients of the regression model since the variables are scaled to similar units centered to the mean of 0 and SD of 1. [22] In all analyses, a 95% confidence interval (CI) with 5% precision was assumed.

## Result

### Patient characteristics

A total of 423 patients participated in the study. The average age of patients was 52.7 (± 15.4) years. The majority of the study participants had no formal education (55.7%, *N* = 214), and this was also reflected in the treatment burden groups with a higher proportion of patients reporting no formal education. Furthermore, most of the patients (78.6%, *N* = 302) had two comorbidities at the time of the study. More than three-fourth (77.8%, *N* = 329) of the study participants had a high treatment burden (Table 1).

**Table 1:** Sociodemographic characteristics stratified by the level of treatment burden

Variable		No-burden (*N* = 9)	Low-burden (*N* = 17)	Medium-burden (*N* = 68)	High-burden (*N* = 329)	Total (*N* = 423)
Gender n (%)	Female	6 (66.7)	9 (52.9)	33 (48.5)	173 (52.6)	221 (52.2)
Age	Years (mean ± SD)	50.6 (± 14.4)	41.6 (± 13.2)	47.0 (± 14.0)	54.5 (± 15.4)	52.7 (± 15.4)
Education n (%)	No formal education	6 (66.7%)	7 (41.2%)	23 (33.8%)	178 (54.1%)	214 (50.6)
	Primary education	3 (33.3%)	1 (5.9%)	13 (19.1%)	69 (21.0%)	86 (20.3)
	Secondary education	0 (0.0%)	3 (17.6%)	13 (19.1%)	40 (12.2%)	56 (13.2)
	Tertiary education	0 (0.0%)	6 (35.3%)	19 (27.9%)	42 (12.8%)	67 (15.8)
Marital status n (%)	Single	2 (22.2%)	5 (29.4%)	8 (11.8%)	28 (8.5%)	43 (10.2)
	Married	7 (77.8%)	10 (58.8%)	47 (69.1%)	215 (65.3%)	279 (65.9)
	Divorced	0 (0.0%)	1 (5.9%)	7 (10.3%)	22 (6.7%)	30 (7.1)
	Widowed	0 (0.0%)	1 (5.9%)	6 (8.8%)	64 (19.5%)	71 (16.8)
Number of Co-morbidities	Two	5 (55.6%)	13 (76.5%)	58 (85.3%)	226 (68.7%)	302 (71.4)
	Three	2 (22.2%)	4 (23.5%)	10 (14.7%)	93 (28.3%)	109 (25.8)
	Four	2 (22.2%)	0	0	10 (0.03%)	12 (2.8)

### Comorbidities and medications

Hypertension (65.2%, *N* = 276) was the most common comorbidity followed by diabetes mellitus (DM) (39.5%, *N* = 167) and heart failure (19.1%, *N* = 8) (Supplementary Fig. 1). Diuretics (54.6%, *N* = 231), angiotensin-converting enzyme inhibitors (ACEIs) (43.7%, *N* = 184), calcium channel blockers (CCBs) (35.7%, *N* = 151), and beta-blockers (32.4%, *N* = 137) were the most commonly prescribed medications (Supplementary Fig. 2).

### Treatment burden and HRQoL

Responses to the MTBQ 10-item questions are depicted in Table 2. The mean global MTBQ score was 39.35 (± 22.16), indicating a high treatment burden overall. The overall mean scores for the EQ-5D index and EQ-VAS were 0.83 (± 0.20) and 67.32 (± 18.51), respectively. Significant differences were observed in the mean EQ-5D-Index (F [2, 81.88] 33.1; *P* < 0.001) and EQ-VAS score (F [2, 75.48] 72.87; *P* < 0.001) as depicted in Tables 3 and 4. Post-hoc analyses revealed significant mean differences in EQ-VAS scores across the treatment burden groups, as well as in EQ-5D index between the no/low treatment burden and high treatment burden, and between the medium treatment burden and high treatment burden (see Tables 3 and 4). Patients’ responses to the five dimensions are also presented in supplementary Table 1.

**Table 2:** Responses to multimorbidity treatment burden questionnaire

Questions	Not difficult *N* (%)	A little difficult *N* (%)	Quite difficult *N* (%)	Very difficult *N* (%)	Extremely difficult *N* (%)
Taking lots of medications	84 (19.86)	76 (17.97)	108 (25.53)	97 (22.93)	58 (13.71)
Remembering how and when to take medication	100 (23.64)	82 (19.89)	104 (24.59)	96 (22.70)	41 (9.69)
Collecting prescription medication	82 (19.39)	90 (21.28)	121 (28.61)	102 (24.11)	28 (6.62)
Monitoring your medical conditions (eg, checking your blood pressure or blood sugar, monitoring your symptoms, etc.)	75 (17.73)	128 (30.26)	119 (28.13)	83 (19.62)	18 (4.26)
Arranging appointments with health professionals	62 (14.66)	132 (31.21)	137 (32.39)	75 (17.73)	17 (4.02)
Seeing lots of different health professionals	65 (15.37)	118 (27.90)	131 (30.97)	90 (21.28)	19 (4.49)
Attending appointments with health professionals (eg, getting time off work, arranging transport, etc.)	82 (19.39)	126 (29.79)	103 (24.35)	75 (17.73)	37 (8.75)
Obtaining clear and up-to-date information about your condition	136 (32.15)	135 (31.91)	88 (20.80)	43 (10.17)	21 (4.96)
Making recommended lifestyle changes (eg, diet and exercise)	169 (39.95)	104 (24.59)	81 (19.15)	53 (12.53)	16 (3.78)
Having to rely on help from family and friends	188 (44.44)	70 (16.55)	80 (18.91)	54 (12.77)	31 (7.33)

**Table 3:** EQ-5D Index stratified by the level of treatment burden

Group	*n*	Mean	*SD*	*Welch F*	*P value*	Games-Howell post hoc analysis			
						No/Low burden		Medium burden	
						Mean difference (95% CI)	*P* value	Mean difference (95% CI)	*P* value
No/Low burden	26	0.95	0.09	33.10	< 0.001				
Medium burden	68	0.92	0.09			− 0.04 (− 0.09 to − 0.02)	0.22		
High burden	329	0.81	0.22			− 0.15 (− 0.20 to − 0.10)	**< 0.001**	− 0.11 (− 0.15 to − 0.07)	**< 0.001**

Bold represents a *p*-value < 0.05

*CI* Confidence Interval, *EQ-5D* Euroqol-five dimensions, *SD* Standard deviation.

**Table 4:** EQ-VAS score stratified by the level of treatment burden

Group	*n*	Mean	*SD*	*Welch F*	*P value*	Games-Howell post hoc analysis			
						No/Low burden		Medium burden	
						Mean difference (95% CI)	*P* value	Mean difference (95% CI)	*P* value
No/Low burden	26	87.10	8.85	72.87	< 0.001				
Medium burden	68	76.50	13.50			− 10.64 (− 16.35 to − 4.94)	**< 0.001**		
High burden	329	63.90	18.40			− 23.26 (− 28.13 to − 18.38)	**< 0.001**	− 12.61 (− 17.17 to − 8.05)	**< 0.001**

Bold represents a *p*-value < 0.05

*CI* Confidence Interval, *EQ-VAS* European quality of life-Visual Analogue Scale, *SD* Standard Deviation

An inverse correlation was observed between the global MTBQ score and the EQ-5D outcomes, including the EQ-5D index and EQ-VAS score. A strong correlation was found between the global MTBQ score and the EQ-VAS score (rho = − 0.51, *P* < 0.001), as well as a moderate correlation between the global MTBQ score and the EQ-5D index (rho = − 0.40, *P* < 0.001). In the multivariate linear regression model, adjusting for standardized age, sex, number of morbidities, and polypharmacy, every one SD increase in the global MTBQ score (i.e., 22.16) was associated with a decline of 0.08 in the EQ-5D index (β − 0.38, 95%CI -0.48, − 0.28, multiplied by the SD of EQ-5D index 0.20). Similarly, a one SD increase in the global MTBQ score reduced the EQ-VAS score by 9.4 (β − 0.51, 95%CI − 0.60, − 0.42, multiplied by the SD of the EQ-VAS score 18.51). Another factor that showed statistically significant associations with the EQ-5D index and EQ-VAS scores was standardized age (Tables 5 and 6).

**Table 5:** Association between patient characteristics and EQ-5D index score

	Univariate			Multivariate┼		
	β (95% CI)	SE	*P* value	β (95% CI)	SE	*P* value
Global MTBQ score	− 0.38 (− 0.47, − 0.29)	0.05	** < 0.0001**	− 0.38 (− 0.48, − 0.28)	0.05	** < 0.0001**
Age (years)	− 0.008 (− 0.014, − 0.0015)	0.003	**0.016**	− 0.11 (− 0.21, − 0.01)	0.05	**0.03**
Sex (Male)	− 0.10 (− 0.30, 0.10)	0.10	0.32	− 0.08 (− 0.28, 0.11)	0.10	0.41
Number of morbidities (≥ 3)	− 0.21 (− 0.43, 0.003)	0.11	0.05	− 0.15 (− 0.37, 0.08)	0.12	0.20
Polypharmacy	− 0.28 (− 0.53, − 0.03)	0.13	**0.03**	− 0.21 (− 0.48, 0.06)	0.14	0.12

Bold represents a *p*-value < 0.05

*CI* confidence interval, *SE* standard error, *EQ-5D* European Quality of life-five dimensions.

^┼^adjusted for standardized age, sex, number of morbidities, and polypharmacy.

**Table 6:** Association between patient characteristics and EQ-VAS score

	Univariate			Multivariate┼		
	β (95% CI)	SE	*P* value	β (95% CI)	SE	*P* value
Global MTBQ score	− 0.53 (− 0.61, − 0.44)	0.04	** < 0.0001**	− 0.51 (− 0.60, − 0.42)	0.05	**< 0.0001**
Age (years)	− 0.014 (− 0.02, − 0.008)	0.003	** < 0.0001**	− 0.21 (− 0.31, − 0.12)	0.05	** < 0.0001**
Sex (Male)	− 0.05 (− 0.24, 0.15)	0.10	0.620	− 0.01 (− 0.20, 0.18)	0.10	0.90
Number of morbidities (≥ 3)	− 0.27 (− 0.49, − 0.06)	0.11	**0.01**	− 0.22 (− 0.43, 0.005)	0.11	0.06
Polypharmacy	− 0.30 (− 0.55, − 0.04)	0.13	**0.02**	− 0.17 (− 0.44, 0.09)	0.13	0.19

Bold represents a *p*-value < 0.05

*CI* confidence interval, *SE* standard error, *EQ* European Quality of life, *VAS* visual analogue scale.

┼adjusted for standardized age, sex, number of morbidities, and polypharmacy.

## Discussion

The present study examined the treatment burden and HRQoL among patients with multimorbidity in a low-income country setting. As patients with multimorbidity are exposed to a number of medications to manage the diseases, this situation may lead to an overwhelming treatment burden on the patients. The current study showed this scenario as a high treatment burden was captured in the overall patient population. One of the impacts of multimorbidity and treatment burden is the decline in patients’ HRQoL. This trend was also observed in the present study as we observed a pattern of lower HRQoL in relation to a higher treatment burden.

Due to the limited number of studies available, it is challenging to put the current findings into perspective. The findings of the present study showed that the majority (77.8%) of patients with multimorbidity experience high treatment burden. In comparison, 26.6% of study participants reported a high treatment burden in the United Kingdom (UK) [22]. The difference can be attributed to differences in age between the two studies and the distinct structures of the healthcare systems in each country. The patients in the current study were relatively younger (53 years old) than the UK study participants (74 years old) [22]. Previous studies reported similar scenarios where younger patients tend to develop a higher treatment burden as compared to older patients [22, 27]. Possible explanations for this phenomenon include the fact that younger patients are often employed and must juggle work, personal life, hospital visits, and taking multiple medications. The health care system of Ethiopia is significantly different than that of the UK, and this difference plays a pivotal role in patients’ treatment outcomes and HRQoL. In Ethiopia, patients need to go through various hurdles to access health care facilities. Most hospitals are further away from the rural part of Ethiopia and patients need to travel long distances to attend medical services. Furthermore, unlike the UK, where most medications are covered through the universal healthcare system called the National Health Service [28], out of pocket is still the predominant way of covering medical expenses in Ethiopia [29]. This situation ultimately exposes patients to financial hardship, making it difficult for them to afford medications [29]. Current efforts are taken to provide universal health coverage through social health insurance, community-based health insurance, and private health insurance schemes. [29] However, the financing of these schemes and coverage is still in the infancy stage [29]. Another factor that may have contributed to a high treatment burden is the low health literacy in the present study setting, as poor health literacy was associated with increased treatment burden. [8]

A significant negative correlation was found between the global MTBQ and the EQ-5D-index, in line with previous findings.[22, 30] Significant differences were also observed in the mean EQ-5D-index and EQ-VAS score among the treatment burden strata. Post-hoc analyses demonstrated significant differences among the treatment burden groups. Additionally, patients who reported a high treatment burden also reported poorer HRQoL in all EQ-5D domains/dimensions. This implies that patients experiencing a high treatment burden also face challenges in fulfilling their daily routines and social responsibilities.

To the best of the authors’ knowledge, this is the first study to capture the relationship between treatment burden and HRQoL in Ethiopian patients with multimorbidity. Literature is also limited in the LMICs setting and this study will provide useful data in this part of the world. Additionally, the study had a sufficient sample size and used two validated tools to measure treatment burden and HRQoL. Nevertheless, the present study has some limitations. The study was conducted in a single setting targeting patients with chronic diseases. Also, the study used a cross-sectional study design and causal associations could not be established.

This study shows important information about treatment burden and health-related quality of life, and the interaction between these two factors. Previous studies have rarely assessed treatment burden and health-related quality of life in the low and middle-income settings. Hence, this study provides vital data to understand patient’s experience, and the need for the health system to balance treatment exposure and maintaining a good quality of life. However, the study is a single center cross-sectional study, and the findings may not be generalizable beyond the study setting; thus, further studies are needed to corroborate the current findings.

## Conclusion

Treatment burden was found to be very common among patients with multimorbidity, and the findings indicated an inverse association between HRQoL and global MTBQ score. Health care providers should be mindful of balancing treatment exposure with patients’ HRQOL, and the health care system should enhance its capacity to address this significant challenge. Future multicenter studies with prospective follow-up are recommended to validate the current findings.

### Supplementary Information

Below is the link to the electronic supplementary material.

Supplementary file1 (DOCX 233 KB)

Supplementary file2 (PDF 131 KB)

Supplementary file3 (PDF 114 KB)

## Data Availability

The data that support the findings of this study are not openly available and are available from the corresponding author upon reasonable request.
