# Supplementary material for: Treatment burden and health-related quality of life of patients with multimorbidity: a cross-sectional study
Source: Qual Life Res. 2023 Jul 5;32(11):3269–77. doi: 10.1007/s11136-023-03473-3 (PMC10522511; doi:10.1007/s11136-023-03473-3)
Supplement: Supplementary file 1 — Supplementary file1 (DOCX 233 KB) [file 11136_2023_3473_MOESM1_ESM.docx]

Supplementary Information

[Supplementary table 1. 2](#_Toc135730095)

[Supplementary fig 1. 3](#_Toc135730096)

[Supplementary fig 2. 4](#_Toc135730097)

Supplementary table 1. EQ-5D dimensions stratified by the level of treatment burden

| Variable |  | Total  (N= 423) | No burden (N=9) | Low burden (N=17) | Medium burden (N=68) | High burden (N=329) |
| --- | --- | --- | --- | --- | --- | --- |
| Mobility n (%) |  |  |  |  |  |  |
|  | No problems | 217 (51.3) | 9 (100.0) | 14 (82.4) | 47 (69.1) | 147 (44.7) |
|  | Slight problems | 108 (25.5) | 0 | 2 (11.8) | 12 (17.6) | 94 (28.6) |
|  | Moderate problems | 79 (18.7) | 0 | 1 (5.9) | 9 (13.2) | 69 (21) |
|  | Severe problems | 18 (4.3) | 0 | 0 | 0 | 18 (5.5) |
|  | Unable | 1 (0.2) | 0 | 0 | 0 | 1 (0.3) |
| Self-care |  |  |  |  |  |  |
|  | No problems | 226 (53.4) | 9 (100.0) | 14 (82.1) | 43 (63.2) | 160 (48.6) |
|  | Slight problems | 108 (25.5) | 0 | 2 (11.8) | 18 (26.5) | 88 (26.7) |
|  | Moderate problems | 69 (16.3) | 0 | 1 (5.9) | 7 (10.3) | 61 (18.5) |
|  | Severe problems | 20 (4.7) | 0 | 0 | 0 | 20 (6.1) |
|  | Unable | 0 | 0 | 0 | 0 | 0 |
| Usual activities |  |  |  |  |  |  |
|  | No problems | 158 (37.4) | 9 (100.0) | 13 (76.5) | 32 (47.1) | 104 (31.6) |
|  | Slight problems | 135 (31.9) | 0 | 3 (17.6) | 23 (33.8) | 109 (33.1) |
|  | Moderate problems | 107 (25.3) | 0 | 1 (5.9) | 13 (19.1) | 93 (28.3) |
|  | Severe problems | 23 (5.4) | 0 | 0 | 0 | 23 (7.0) |
|  | Unable | 0 | 0 | 0 | 0 | 0 |
| Pain/Discomfort |  |  |  |  |  |  |
|  | No pain | 133 (31.4) | 9 (100.0) | 13 (76.5) | 35 (51.5) | 76 (23.1) |
|  | Slight pain | 130 (30.7) | 0 | 3 (17.6) | 18 (26.5) | 109 (33.1) |
|  | Moderate pain | 116 (27.4) | 0 | 1 (5.9) | 13 (19.1) | 102 (31.0) |
|  | Severe pain | 43 (10.2) | 0 | 0 | 2 (2.9) | 41 (12.5) |
|  | Extreme pain | 1 (0.2) | 0 | 0 | 0 | 1 (0.3) |
| Anxiety/Depression |  |  |  |  |  |  |
|  | Not anxious/depressed | 227 (53.7) | 7 (77.8) | 15 (88.2%) | 47 (69.1) | 158 (48.0) |
|  | Slightly anxious/depressed | 138 (32.6) | 0 | 2 (11.8%) | 21 (30.9) | 115 (35.0) |
|  | Moderately anxious/depressed | 39 (9.2) | 0 | 0 | 0 | 39 (11.9) |
|  | Severely anxious/depressed | 17 (4.0) | 2 (22.2) | 0 | 0 | 15 (4.6) |
|  | Extremely anxious/depressed | 2 (0.5) | 0 | 0 | 0 | 2 (0.6) |


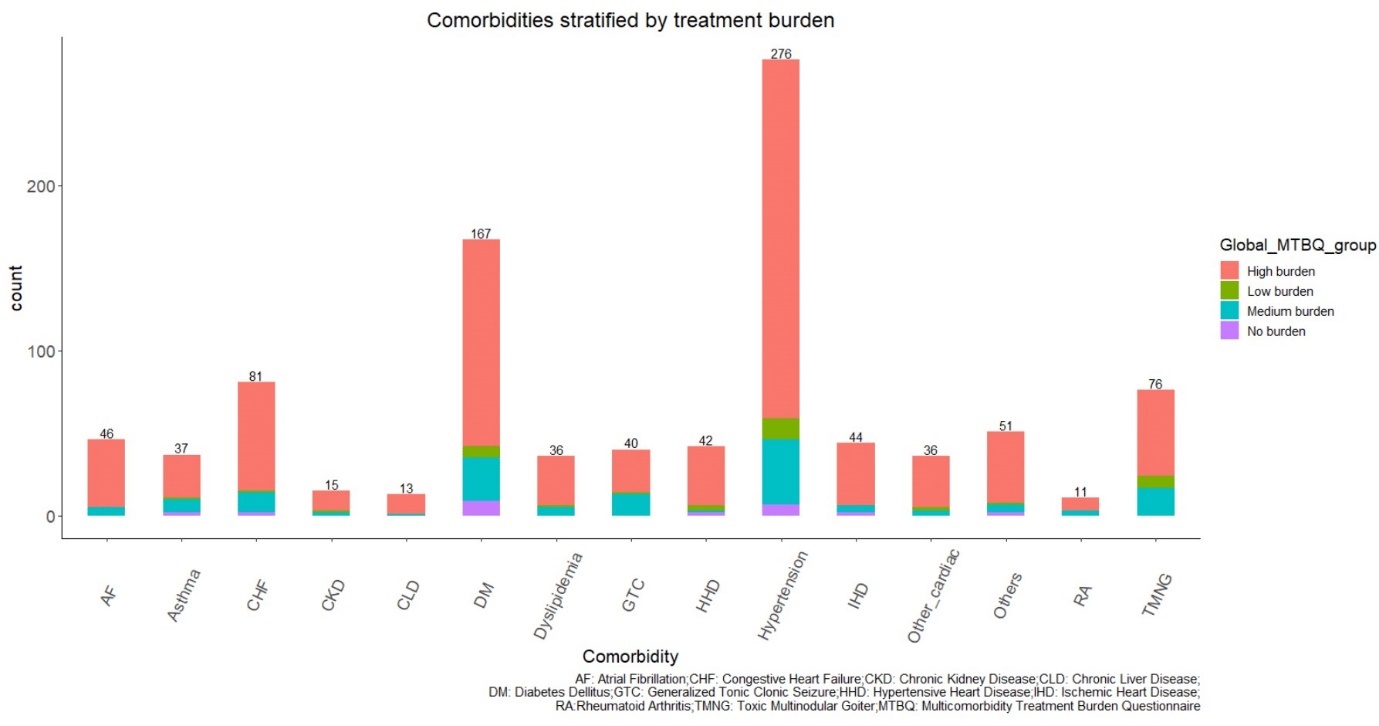


Supplementary fig 1. Comorbidities stratified by treatment burden


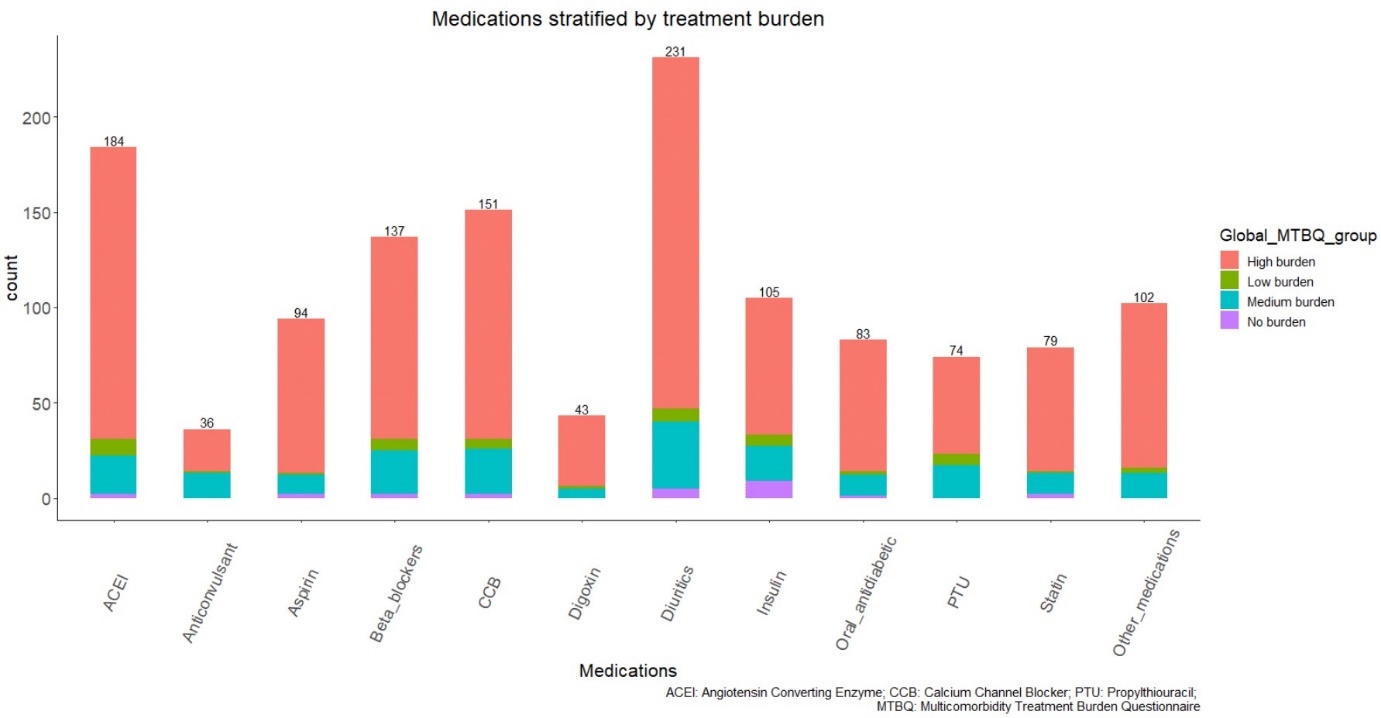


Supplementary fig 2. Medications stratified by treatment burden
