# Supplementary material for: Treatment burden and health-related quality of life of patients with multimorbidity: a cross-sectional study
Source: Qual Life Res. 2023 Jul 5;32(11):3269–77. doi: 10.1007/s11136-023-03473-3 (PMC10522511; doi:10.1007/s11136-023-03473-3)
Supplement: Supplementary file 2 — Supplementary file2 (PDF 131 KB) [file 11136_2023_3473_MOESM2_ESM.pdf]

እድሜ_______ ጾታ _____
የት/ት ደረጃ_________________የጋብቻ ሁኔታ ___________ስራ ________________

ይህ መጠይቅ ያለብዎትን የጤና ሁኔታ እንዲሁም የጤና ሁኔታዎን ለመቆጣጠርና ለማሻሻል የሚያደርጉት ጥረት በእለት ተእለት ህይወትዎ ላይ ያለውን ተፅዕኖ ለማወቅ የተዘጋጀው ነው፡፡

ከዚህ በታች የተዘረዘሩትን ተግባራት በአግባቡ ለማከናወን ምን ያህል እንደሚቸገሩ ይገልፁልን ዘንድ እንጠይቃለን፤ (ምላሽዎን ከእያንዳንዱ ጥያቄዎች ጎን በተቀመጡት ሳጥኖች ዉስጥ ምልክት በማድረግ ይግለፁ፡፡)

	እጅግ በጣም ከባድ	በጣም ከባድ	የተወሰነ ይከብዳል	በትንሹ ይከብዳል	ምንም አይከብድም
1.ብዙ መድሃቶችን መውሰድ					
2.መድሃኒቶችን መቼ እና እንዴት እንደሚወሰድ ማስታወስ					
3.የታዘዙትን መድሃኒቶች ከመድሃኒት ቤት መውሰድ (መግዛት)					
4.የጤንነት ሁኔታዎን መከታተል (የደም ግፊትዎን መለካት፣ የደም የስኳር መጠንዎን እንዲሁም የሚሰማዎትን ነገር መቆጣጠር (መከታተል))					
5.ከጤና ባለሞያዎች ጋር ያለዎትን ቀጠሮ ማመቻቸት					
6.የተለያዩ አይነት የጤና ባለሞያዎችን ማግኘት					
7.ከጤና ባለሙያ ጋር ያለዎትን የህክምና ቀጠሮ መከታተል (ከስራ ቦታ ውጥቶ መምጣትመጓጓዣ/ትራንስፖርት ማመቻቸት. . .)					
8. ስለጤና ሁኔታዎ ግልጽ እና አዳዲስ መረጃዎች ማግኘት					
9በጤና ባለሙያ የተነገረዎትን የአካል ብቃት እንቅስቃሴና የአመጋገብ ስርአት መተግበር					
10. በቤተሰብ (ቤተ ዘመድ) እና ጓደኛ ድጋፍ ላይ ጥገኛ መሆን					

በእያንዳንዱ ርዕስ ስር፣ እባክዎ ዛሬ ያለዎትን ጤንነት በተሻለ ሁኔታ የሚገልጸው አንድ ሳጥን ላይ ምልክት ያድርጉ፡፡

**እንቅስቃሴ**		**ራስን መንከባከብ**	
የመራመድ ችግር የለብኝም		ለመታጠብም ሆነ ለመልበስ ምንም ችግር የለብኝም	
አነስተኛ የሆነ የመራመድ ችግር አለብኝ		ለመታጠብም ሆነ ለመልበስ አነስተኛ የሆነ ችግር አለብኝ	
መጠነኛ የሆነ የመራመድ ችግር አለብኝ		ለመታጠብም ሆነ ለመልበስ መጠነኛ ችግር አለብኝ	
ከባድ የሆነ የመራመድ ችግር አለብኝ		ለመታጠብም ሆነ ለመልበስ ከፍተኛ የሆነ ችግር አለብኝ	
ምንም መራመድ አልችልም		ራሴ ልታጠብም ሆነ ልለብስ አልችልም	

**መደበኛ ተግባራት** *(ለምሳሌ፡- ስራ፣ ትምህርት፣ የቤት ውስጥ ስራ፣ ቤተሰባዊ ወይም የእረፍት ጊዜ ተግባራት)*		**የሕመም ስሜት/ምቾት ማጣት**	
መደበኛ ተግባራቶቼን ያለ ምንም ችግር አከናውናለሁ		የሕመም ስሜትም ሆነ የምቾት ማጣት ስሜት የለኝም	
መደበኛ ተግባራቶቼን ለማከናወን አነስተኛ ችግር አለብኝ		አነስተኛ የሕመም ስሜት ወይም የምቾት ማጣት ስሜት አለኝ	
መደበኛ ተግባራቶቼን ለማከናወን መጠነኛ ችግር አለብኝ		መጠነኛ የሕመም ስሜት ወይም የምቾት ማጣት ስሜት አለኝ	
መደበኛ ተግባራቶቼን ለማከናወን ከፍተኛ ችግር አለብኝ		ከባድ የሕመም ስሜት ወይም የምቾት ማጣት ስሜት አለኝ	
መደበኛ ተግባራቶቼን ለማከናወን አልችልም		የከፋ የሕመም ስሜት ወይም የምቾት ማጣት ስሜት አለኝ	

**ጭንቀት/ድብርት**	
ጭንቀትም ሆነ ድብርት የለብኝም	
አነስተኛ ጭንቀት ወይም ድብርት አለብኝ	
መጠነኛ ጭንቀት ወይም ድብርት አለብኝ	
ከባድ ጭንቀት ወይም ድብርት አለብኝ	
እጅግ ከባድ ጭንቀት ወይም ድብርት አለብኝ	

ሊኖርዎ የሚችለው
በጣም ጥሩው የጤና
ሁኔታ

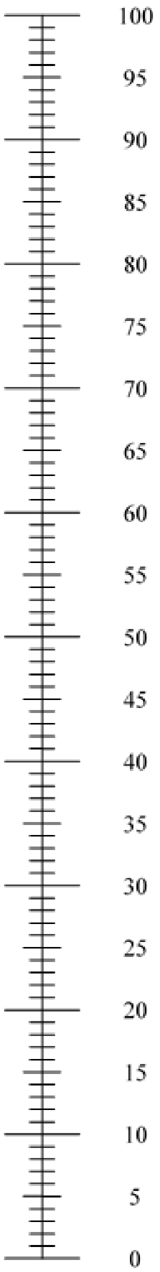

ሊኖርዎ የሚችለው
በጣም መጥፎው
የጤና ሁኔታ

የዛሬ ጤንነትዎ = [ ]

- **ዛሬ** የጤና ሁኔታዎ ምን ያህል ጥሩ ወይም መጥፎ መሆኑን ለማወቅ እንፈልጋለን፡፡
- መለኪያው ከ0 እስከ 100 ድረስ ቁጥሮች አሉት፡፡
- 100 ማለት ሊኖርዎ የሚችለው በጣም ጥሩው የጤና ሁኔታ ነው።
  0 ማለት ሊኖርዎ የሚችለው በጣም መጥፎው የጤና ሁኔታ ነው።
- በመለኪያው ላይ **ዛሬ** ጤንነትዎ ያለበትን ሁኔታ ለማሳየት የ X ምልክት ያድርጉ፡፡
- አሁን፣ከስር ባለው ሳጥን ውስጥ በለመኪያው ላይ ምልክት ያደረጉበትን ቁጥር ይጻፉ፡፡
