# Supplementary material for: Treatment burden and health-related quality of life of patients with multimorbidity: a cross-sectional study
Source: Qual Life Res. 2023 Jul 5;32(11):3269–77. doi: 10.1007/s11136-023-03473-3 (PMC10522511; doi:10.1007/s11136-023-03473-3)
Supplement: Supplementary file 3 — Supplementary file3 (PDF 114 KB) [file 11136_2023_3473_MOESM3_ESM.pdf]

Age_______ Sex _____

Educational status___________Marital status ___________Occupation ________________

We are interested in finding out about the effort you have to make to look after your health and how these impacts on your day-to-day life.

Please tell us how much difficulty you have with the following: (Please tick the box that most applies to you)

	Extremely difficult	Very difficult	Quite difficult	A little difficult	Not difficult	Does not apply
1. Taking lots of medications						
2. Remembering how and when to take medication						
3. Collecting prescription medication						
4. Monitoring your medical conditions (e.g. checking your blood pressure or blood sugar, monitoring your symptoms etc.)						
5. Arranging appointments with health professionals						
6. Seeing lots of different health professionals						
7. Attending appointments with health professionals (e.g. getting time off work, arranging transport etc.)						
8. Obtaining clear and up-to-date information about your condition						
9. Making recommended lifestyle changes (e.g. diet and exercise etc.)						
10. Having to rely on help from family and friends						

Under each heading, please tick the ONE box that best describes your health TODAY.

**Mobility**		**Self-care**	
I have no problems in walking about		I have no problems washing or dressing myself	
I have slight problems in walking about		I have slight problems washing or dressing myself	
I have moderate problems in walking about		I have moderate problems washing or dressing myself	
I have severe problems in walking about		I have severe problems washing or dressing myself	
I am unable to walk about		I am unable to wash or dress myself	

**Usual activities** *(e.g., work, study, housework, family or leisure activities)*	
I have no problems doing my usual activities	
I have slight problems doing my usual activities	
I have moderate problems doing my usual activities	
I have severe problems doing my usual activities	
I am unable to do my usual activities	

**Pain/discomfort**	
I have no pain or discomfort	
I have slight pain or discomfort	
I have moderate pain or discomfort	
I have severe pain or discomfort	
I have extreme pain or discomfort	

**Anxiety /depression**	
I am not anxious or depressed	
I am slightly anxious or depressed	
I am moderately anxious or depressed	
I am severely anxious or depressed	
I am extremely anxious or depressed	

A very good health condition

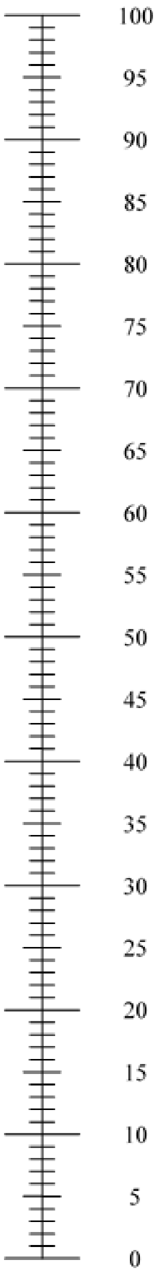

A worst health condition

Your health today = ☐

- We want to assess your health condition today.
- The figure has its own scale from 0- 100.
- 100 implies that you have a very good health condition.
- 0 implies you are experiencing a worst health condition.
- Please show your health status on the figure by ticking on the scales
- Now write the number you have marked on the box above
